# Evaluation of the efficacy of Tai Chi on the cognitive function of patients with mild cognitive dysfunction and research on its mechanism

**DOI:** 10.3389/fnagi.2025.1435996

**Published:** 2025-04-02

**Authors:** Lin Lin, Yao-Xi He, Quan Wen, Jia-Yang Liu, Yu Dai, Yu-Zhe Fei, Hang Li, Chang-Qing Li, Huan Zhou

**Affiliations:** ^1^West China School of Public Health and West China Fourth Hospital, Sichuan University, Chengdu, Sichuan, China; ^2^School of Elderly Health/Collaborative Innovation Center of Sichuan for Elderly Care and Health, Chengdu Medical College, Chengdu, Sichuan, China; ^3^School of Nursing, Chengdu Medical College, Chengdu, Sichuan, China; ^4^Chengdu Eighth People’s Hospital (Geriatric Hospital of Chengdu Medical College), Chengdu, Sichuan, China; ^5^Healthy Aging Research Center, West China-PUMC C.C. Chen Institute of Health, Sichuan University, Chengdu, Sichuan, China

**Keywords:** Tai Chi, MCI, BDNF, cognitive function, PF4

## Abstract

**Introduction:**

Studies have revealed that Tai Chi can enhance cognitive functions among patients with mild cognitive impairment (MCI). However, the precise mechanisms underlying this improvement remain elusive.

**Methods:**

Consequently, we conducted a study involving 54 elderly inpatients with MCI residing in a combined medical and elderly care facility in Chengdu, who were randomly divided into three groups: a control group engaging in daily living activities, a Tai Chi group that performed Tai Chi exercises in addition to control group activities, and a walking group that undertook walking activities as a supplement to the control group regimen. The intervention period lasted for 24 weeks, comprising 12 weeks of exercise and an additional 12 weeks of follow-up. The Montreal Cognitive Assessment (MoCA), Trail Making Test-A (TMT-A), Auditory Verbal Learning Test (AVLT), and biochemical assessments (measuring brain-derived neurotrophic factor, BDNF, and platelet factor 4, PF4) were administered to investigate overall cognitive function, executive function, memory capacity, and changes in serum concentrations of BDNF and PF4 before, after, and during the follow-up period. Data were analyzed using IBM SPSS 26.0, with statistical methods encompassing descriptive analysis, ANOVA, rank-sum test, repeated measures ANOVA, and generalized estimating equations.

**Results:**

Our findings indicated that after 24 weeks of intervention, the Tai Chi group exhibited improvements in cognitive function, executive function, and memory compared to the control group. This enhancement may be attributed to an increased expression of serum BDNF.

**Discussion:**

In conclusion, our study underscores the potential of Tai Chi in ameliorating cognitive function among elderly patients with mild cognitive impairment, thereby offering significant implications for clinical prevention and treatment strategies targeting this condition.

## 1 Introduction

Population aging is the biggest social problem that countries around the world are facing now and even in the next few decades, and according to the United Nations, in 2019, 9% of the world’s population will be over the age of 65, and it is expected that the proportion can reach 16% by 2050 (United Nations, 2019). China entered the aging society at the end of the 20th century, and the rate of population aging and the number of elderly people are the highest in the world, the seventh national census data show that China’s elderly population aged 60 years or older is 200 million, accounting for 13.5% of the total population, and by 2030, China’s elderly population aged 60 years or older is expected to reach 400 million, accounting for 30% of the world’s total elderly population, which indicates that China has entered the deep aging society (Tong, 2021). Population aging also brings with it a rapid increase in age-related disabilities and diseases, and as the world’s population continues to age at an accelerating rate, one new case of dementia is added every three seconds, with about 50 million people currently living with dementia globally, and the number of dementia patients will increase to 139 million by 2050 (Liu et al., 2021). There are about 15.07 million dementia patients in China, accounting for 25.5% of the world’s total dementia population (Rujing et al., 2021). The most common type of dementia is Alzheimer’s disease (AD), which accounts for 50%–75% of all dementia cases (Prince et al., 2014), and the number of AD in China is as high as 9.83 million people, and it is expected that it will reach 30.03 million people in 2050, which will become a major disease and social problem that jeopardizes the health of the population in China (Wang Y. et al., 2019). Mild Cognitive Impairment (MCI) as a transitional stage between normal functioning and dementia, the patients in this stage mainly show forgetfulness, which does not affect the ability of daily life activities, and it is considered to be a key stage in the prevention of AD (Bennett et al., 2002), and it may serve to delay the development of AD if interventions can be implemented in this stage. In addition, there is no gold standard or drug for the treatment of MCI (Tricco et al., 2013), therefore, non-pharmacological interventions are gaining more and more support (Rodakowski et al., 2015), and exercise is a promising non-pharmacological therapy. Many governments and non-governmental organizations have specifically developed guidelines for physical activity in older adults, and the Centers for Disease Control and Prevention (CDC) advocates exercise for older adults, stating that it is essential for healthy aging (Centers for Disease Control and Prevention, 2023). For older adults with gradually declining physical function, aerobic exercises such as table tennis, cycling, swimming, and resistance exercises such as the use of elastic bands, dumbbells, and sandbags are difficult to adhere to because they are more intense, which is of less interest to older adults, and the participation rate is not high; in addition, these exercises are often limited by venues and equipment (Maltais et al., 2019). As Tai Chi, a traditional exercise cherished by the elderly, gains widespread popularity, an increasing number of studies have delved into its role in enhancing cognitive functions among patients with MCI and Alzheimer’s disease (AD) (Huang et al., 2022; Jia et al., 2023; Wang et al., 2018; Wayne et al., 2014; Zou et al., 2019). For instance, an experimental randomized controlled trial comparing the effects of 24-form Yang-style Tai Chi versus similar-intensity conventional exercise on the overall cognitive abilities of MCI patients revealed that Tai Chi appeared to facilitate earlier and more pronounced improvements in cognitive flexibility (Yu et al., 2022). Additionally, a systematic review has shown that Tai Chi practice, spanning from 12 weeks to 1 year, can yield small to moderate clinically relevant enhancements in the overall cognitive functioning of elderly individuals with cognitive impairment, when compared to non-intervention control groups or other active interventions such as adaptive physical activities, health education, stretching and relaxation exercises, and walking (Zhou et al., 2022). However, the majority of these studies have primarily focused on changes in cognitive test scores, with scarce investigations into associated blood-based biological markers. Consequently, there is a pressing need to conduct in-depth research into the neurobiological mechanisms underlying Tai Chi’s potential to ameliorate cognitive functions in MCI patients.

In summary, the prevalence of MCI is high, and the likelihood of progression to Alzheimer’s disease (AD) is substantial. The majority of individuals with MCI experience sleep disturbances and emotional disorders, such as anxiety and depression, which expedite the progression of MCI into AD. Consequently, early intervention is paramount in managing mild cognitive decline. Tai Chi, a form of exercise potentially harboring significant potential to enhance cognitive function in MCI patients, has yet to reach a consensus in existing research outcomes. Moreover, there is a dearth of studies examining the impact of Tai Chi on hematological indicators, and the underlying biological mechanisms responsible for its cognitive benefits remain elusive. Studies have indicated that exercise can elevate gene expression and protein levels of brain-derived neurotrophic factor (BDNF) in various regions of the brain and periphery. This augmentation stimulates angiogenesis, fosters the growth of new blood vessels and neurons, and mitigates inflammation (Stillman et al., 2016). Furthermore, recent animal experiments have revealed that platelet factor 4 (PF4) significantly reduces inflammatory responses in aged mice, thereby improving their cognitive function (Schroer et al., 2023). Notably, exercise activates PF4, which promotes the proliferation of hippocampal progenitor cells in elderly mice, restoring cognitive function and enhancing learning and memory capabilities (Leiter et al., 2023). These findings collectively suggest that PF4 positively influences cognitive function in aged mice, hinting at its potential beneficial effects in elderly humans with declining cognitive abilities. Therefore, this study targeted patients with MCI and aimed to explore the effects of a 12-week Tai Chi intervention, compared to a walking group and standard care, on cognitive function, serum brain-derived neurotrophic factor (BDNF), and platelet factor 4 (PF4) levels in MCI patients.

## 2 Materials and methods

### 2.1 Study design and randomization

This study was conducted as a randomized controlled trial, where randomization was carried out by a dedicated statistical analyst who was not involved in the research process. The analyst utilized Excel to generate random numbers and produce allocation cards, which were then enclosed in sealed envelopes. The envelopes were opened sequentially as the participants entered the trial, and allocations to specific groups were made according to the instructions on the allocation cards. The study group was randomly divided into a Tai Chi group, a walking group, and a control group at a 1:1:1 ratio. Importantly, the participants remained blinded to their group assignment, and similarly, the outcome assessors and data analysts were also unaware of the group allocations.

### 2.2 Participants

From December 2022 to February 2023, elderly inpatients who voluntarily participated in cognitive function screening were recruited from a medical care facility in Chengdu. Through one-on-one interviews, general information such as the subjects’ age, educational background, pre-retirement occupation, and medical history was collected. Subsequently, the participants underwent comprehensive assessments utilizing the Montreal Cognitive Assessment (MoCA), Mini-Mental State Examination (MMSE), and activities of daily living (ADL) questionnaires, which took approximately 20 to 30 min to complete.

The screening criteria for middle-aged and elderly MCI patients in this study were (Nasreddine et al., 2005): age ≥ 60 years; A 1-year decline in subjective cognitive function as reported by the participants or those in the know (family, friends, doctors, etc.); The total score of the Montreal Cognitive Function Assessment Scale (MoCA) is < 26 points (if the number of years of education at the time of scale assessment is less than 12 years, 1 point is added to the score); ADL (Activity of Daily Living Scale Assessment) score < 26 points (Lawton and Brody, 1969); No dementia, MMSE score > 24 points (primary school: > 20 points; Illiterate: > 17 points). Then, the results of the questionnaire were sent to the Department of Neurology, and the outpatient doctors of the department of neurology diagnosed the patients according to the diagnostic criteria for MCI formulated by the Chinese Alzheimer’s Disease Association.

(1) Inclusion criteria

a. Age requirement: Participants must be aged 60 years or older.
b. Diagnosis confirmation: Individuals must have undergone screening and been diagnosed with MCI.
c. Communication abilities: Candidates must possess the capacity for verbal communication, as well as the ability to read simple texts and write basic sentences.
d. Informed consent: Subjects must have agreed to participate voluntarily and signed an informed consent form acknowledging their full understanding and willingness to participate.
e. Study engagement and hospital stay: Participants must be willing to cooperate with the study’s requirements and have an anticipated hospital stay of six months or longer.

(2) Exclusion criteria

a. Concurrent neurological conditions: Patients with other neurological disorders associated with cognitive impairment are excluded.
b. Current cognitive therapy: Individuals currently undergoing cognitive therapy are not eligible.
c. Chronic mobility-impairing conditions: Those with chronic illnesses that hinder physical mobility are not included.
d. Uncontrolled hypertension: Patients with hypertension that is not adequately controlled are excluded.
e. Sensory and communication barriers: Individuals with visual or hearing impairments that significantly impede communication are not suitable for the study.
f. Participation in other trials: Subjects currently enrolled in other research studies are ineligible.
g. Psychiatric conditions: Patients with psychiatric disorders, including major depressive disorder, severe anxiety, schizophrenia, and the like, are excluded.

(3) Dropout, termination, and exclusion criteria

a. Adverse events during exercise: Participants who experience adverse events, such as severe falls or injuries, during the exercise intervention may be withdrawn from the study.
b. Lack of continuation: Subjects who express unwillingness to continue participating in the research will be discontinued.
c. Deviation from intervention protocol: Those who fail to comply with the prescribed intervention protocol, thereby compromising the authenticity of study results, may be excluded from the analysis.

**FIGURE 1:**
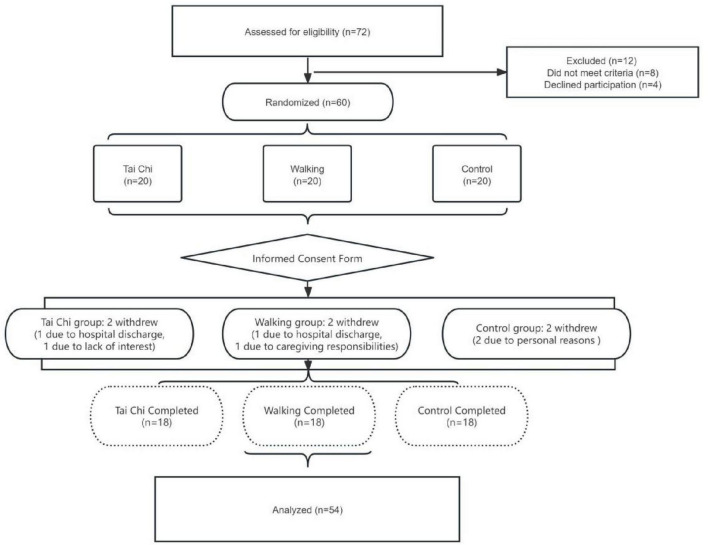
Research flowchart.

The following is the flowchart of this study as shown in Figure 1.

### 2.3 Sample size

The sample size for this study was calculated using the formula for comparing means across multiple groups, with the primary basis being the Montreal Cognitive Assessment (MoCA) scores and supported by relevant literature (Xiao, 2022). The calculated parameters were as follows: mean (X) = 25.386, sum of squared standard deviations (ΣSi^2^/g) = 7.4697/3 = 2.4899, sum of squared deviations from the mean (Σ(Xi–X)^2^) = 2.300088, number of groups (g) = 3, two-sided significance level (α) = 0.05, type II error rate (β) = 0.10, degrees of freedom (V1) = g−1 = 2, and degrees of freedom (V2) = ∞. By referencing the critical value table, the ψ value was determined to be 2.52. Based on the “sample size estimation for comparing means across multiple groups,” the required sample size per group was estimated to be between 14 and 16. To account for a potential 20% dropout rate, the final sample size was set to a minimum of 20 participants per group, resulting in a total sample size of at least 60 participants. The specific formula used for the calculation is as follows:

$$N_{1}=N_{2}=N_{3}={\text{ψ}}^{2}(\Sigma S_{i}^{2}/g)/[\text{Σ}{\left(-Xi--X\right)}^{2}/(g-1)]$$

### 2.4 Ethics statement

This study was ethically reviewed by the Biomedical Ethics Committee of the trial research unit (Chengdu Eighth People’s Hospital). The trial was conducted in accordance with the ethical principles of voluntariness, harmlessness and confidentiality. The researchers informed the subjects about the purpose, methods, benefits and risks of the study and signed an informed consent form. The study protocol was approved by the Ethics Committee (Batch No.: 2023-CBYEC-005) and successfully obtained the registration certification from China Clinical Trial Registry (CCTR), whose registration code is: ChiCTR2400080046.

### 2.5 Interventions

#### 2.5.1 Control group

Conduct routine medical care and maintain daily life activities, mainly including: open the windows and ventilate the sick room twice a day to maintain air circulation, advise patients to eat a light diet, easy to digest, and avoid spicy and stimulating food. The researchers did not interact with the participants in the control group beyond providing routine hospital care.

#### 2.5.2 Tai Chi group

On the basis of the routine activities of the control group, Li et al. (2003) from Oregon Research Institute of the United States compiled eight styles of Tai Chi for exercise, which is simple and easy to learn, with refined content, complete and scientific extraction of the essence of Tai Chi, retaining the basic boxing theory of Tai Chi, inheriting the essence of Tai Chi strokes, and taking the continuous lunge as the main change in the steps, with a rounded and coherent movement, which is suitable for elderly people who do not have a sports foundation to practise. The movements are round and coherent, suitable for the elderly who have no exercise foundation to practice, and it is an easy set of boxing for people who are new to Tai Chi. The eight Tai Chi movements include: starting posture, wild horse parting mane, cloud hand, single whip, inverted curling arm, knee-wrapping step, Jade Maiden shuttle, bird’s tail, cross hand, and closing posture.

According to the actual situation of the study participants during their stay in the hospital, the group practice time was set at 15:30–16:00. The single-session Tai Chi exercise intervention program consisted of a preparation phase, a practice phase, and a recovery phase. The preparation and recovery phases consisted of preparatory and organizing activities, including active stretching and breathing exercises; the practice phase consisted of eight styles of Tai Chi. The entire Tai Chi practice process was completed by one Tai Chi instructor and two observers. During the intervention process, the primary subject conducted weekly interviews with the subjects so as to be able to promptly identify and solve the problems encountered by the subjects in practicing Tai Chi exercises and to urge the subjects to perform Tai Chi exercises. The Tai Chi training lasted for 12 weeks, 3 times a week, and each exercise time was 30 min (including 5 min warm-up, 20 min Tai Chi exercise, and 5 min relaxation and organization). Follow-up was conducted for another 12 weeks. During the 12-week follow-up period, there was no interaction between the Tai Chi instructors and the participants.

#### 2.5.3 Walking group

In addition to routine hospital care, a set of muscle stretching and conditioning walking exercises developed by a rehabilitation therapist. It included warm-up static stretching exercises, i.e., lateral neck flexion, anterior cross-arm stretching, posterior neck triceps stretching, and calf stretching; then flat-ground walking exercise, with the intensity of the walking exercise basically maintained in the interval of 55% to 65% of the maximal heart rate, with the maximal heart rate = (220–age), and finally cooling stretching exercise, consistent with the warm-up static stretching exercises. During the intervention, the researcher conducted weekly interviews with the subjects to be able to identify and address any difficulties encountered by the study subjects during the walking exercise. Walking training was conducted independently by individuals from 15:30 to 16:00 for 12 weeks, 3 times per week, with 30 min of walking time per session, (including 5 min of warm-up, 20 min of walking exercise, and 5 min of relaxation and finishing). Follow-up was conducted for another 12 weeks.

### 2.6 Collection of study data

#### 2.6.1 Outcome measures

MoCA, AVLT and TMT-A scales were assessed by two data researchers who were professionally trained to evaluate all patients included in the study at pre-intervention, post-intervention, and follow-up; the two data researchers did not participate in the intervention trial to maintain objectivity in the evaluation.

The Montreal Cognitive Assessment (MoCA) comprehensively assesses multiple cognitive domains, encompassing attention, executive function, memory, language, visuospatial skills, abstract thinking, calculation abilities, and orientation. Its sensitivity in detecting MCI stands at an impressive 90%, with a specificity of 87%, a stable test-retest reliability of 0.92, and a Cronbach’s alpha coefficient of 0.83 for standardized items, demonstrating robust reliability (Nasreddine et al., 2005). Its positive and negative predictive values for MCI were good (89% and 91%, respectively) (Julayanont et al., 2017).

The Auditory Verbal Learning Test (AVLT), specifically designed to evaluate memory capacity among Chinese mainland residents by Guo et al. (1993), boasts a Cronbach’s α coefficient of 0.99 and a test-retest reliability ranging from 0.87 to 0.94 over a three-month interval, ensuring the stability of the assessment. The AVLT comprises 12 words, subjected to three consecutive readings followed by immediate, delayed, and recognition memory tests, providing a comprehensive evaluation of memory proficiency. The cumulative score from all learning trials comprehensively reflects the subject’s memory level.

The Trail Making Test-A (TMT-A), a classic neuropsychological tool for assessing attention and visual search abilities, requires participants to sequentially connect numbers 1 through 25, with shorter completion times indicating stronger executive function. This test exhibits a sensitivity of 69% and a specificity of 88% in identifying executive dysfunction (Guo and Hong, 2013).

Brain-Derived Neurotrophic Factor (BDNF), a pivotal neuronal nutrient, was first discovered in pig brains by Barde et al. (1982). BDNF and its receptors are widely distributed throughout the nervous system, with particularly high concentrations in the cerebral cortex, hippocampus, and amygdala. By fostering neuronal differentiation, growth, and participating in neuronal repair processes, BDNF is crucial for neural system health (Zahid et al., 2013). This study employs a human BDNF ELISA kit to accurately measure BDNF levels in subjects’ serum.

Platelet Factor 4 (PF4), a cytokine released by platelets, plays a pivotal role in platelet activation and thrombosis. Beyond inhibiting vascular endothelial cell growth and modulating platelet aggregation, PF4 also exhibits antibacterial, antiviral properties, and participates in inflammatory responses, immune regulation, and angiogenesis (Park et al., 2023). Utilizing a human PF4 ELISA kit[, this study quantitatively analyzes PF4 levels in subjects’ serum.

#### 2.6.2 Laboratory data

Early in the morning, before and after the intervention, reviewers collected laboratory samples of BDNF and PF4 from fasting participants. Blood samples (5 mL each) were obtained using plain serum tubes. These samples were allowed to sit at room temperature for 15 min before being centrifuged at 3,000 revolutions per minute in the testing department’s centrifuge. Subsequently, the upper serum layer was transferred into appropriately labeled test tubes, each containing 200 microliters. These test tubes were then stored in a refrigerator at −80°C. Upon completion of specimen collection from all patients, the samples were transported to a testing company for analysis.

### 2.7 Statistical analysis

The general information and scale data of this study were statistically analyzed using IBM SPSS26.0 software. Measurement data were expressed as mean ± standard deviation, and measurement data were first analyzed for normality and variance alignment of the data using the Shapiro–Wilk test and Levene test, and measures that met normal distribution and variance alignment were analyzed using analysis of variance (ANOVA), and multiple comparisons were performed when the results showed statistical differences, and Dunnett method was used when the variance was aligned. Measures that did not conform to normal distribution and variance laxity were analyzed using the Kruskal–Wallis rank sum test, and multiple comparisons were performed using the Dunn–Bonferroni method when the results showed a statistical difference. Count data were expressed as rates (%) using the chi-square test or Fisher’s exact test. The test level was set at α = 0.05, and *p* < 0.05 was considered a statistically significant difference. Differences in outcome indicators between groups over time during the intervention were assessed using generalized estimating equations.

## 3 Results

### 3.1 Baseline characteristics of the subjects

A total of 72 patients with mild cognitive impairment (MCI) were screened for eligibility, of whom 60 met the inclusion criteria and were randomized into the Tai Chi group, walking group, and control group. During the study period, 2 participants from each group dropped out, resulting in 54 MCI patients (18 per group) completing the study. All 54 patients completed scale assessments at baseline, post-intervention, and follow-up. For laboratory indicators, baseline data were collected from all 54 patients, while post-intervention data were obtained from 52 patients (two patients in the control group refused blood draw). The age range of the study subjects was 67–94 years old, with an average age of (84.46 ± 5.961); 66.67% were female and 33.33% were male; the average number of years of education was (10.81 ± 3.905), indicating that the majority of the study subjects had high school diplomas; 33.33% were married, widowed or divorced accounted for 66.67%; no chronic disease accounted for 38.89% and one or more chronic diseases accounted for 61.11%, indicating that most of the elderly patients had one or more chronic diseases; there was a family history of dementia accounted for 18.52%, and there was no family history of dementia accounted for 81.48%, indicating that the vast majority of the elderly did not have a family history of dementia. The pre-intervention general demographic data and outcome indicators of the Tai Chi group, the walking group and the control group were comparable, and the differences were not statistically significant (*p* > 0.05). See Table 1 for specific details.

**TABLE 1:** Comparison of general and baseline data among the three groups of patients (*N* = 54).

	Category	Tai Chi group (*N* = 18)	Walking group (*N* = 18)	Control group (*N* = 18)	Statistical value	*P*
Age (years)		86.17 ± 6.022	85.28 ± 4.650	81.96 ± 6.12	*F* = 2.668	0.079
Sex (cases)	Male	9 (50.0%)	4 (22.2%)	5 (27.8%)	*χ^2^* = 3.500	0.174
	Female	9 (25.0%)	14 (38.9%)	13 (36.1%)		
Years of education (years)		10.89 ± 4.639	10.78 ± 3.135	10.78 ± 4.023	*F* = 0.005	0.995
Number of chronic diseases (number)	0	7 (33.3%)	7 (33.3%)	7 (33.3%)	χ^2^ = 0.158	0.924
	1∼2	6 (35.3%)	6 (35.3%)	5 (29.4%)		
	≥ 3	5 (31.3%)	5 (31.3%)	6 (37.5%)		
Marital status (cases)	Married	7 (38.9%)	4 (22.2%)	7 (38.9%)	χ^2^ = 1.500	0.472
	Widowed or divorced	11 (30.6%)	14 (38.9%)	14 (30.6%)		
Family history of dementia (cases)	Yes	3 (30.0%)	3 (30.0%)	4 (40.0%)	χ^2^ = 0.245	0.885
	No	15 (34.1%)	1,515 (34.1%)	14 (31.8%)		
MoCA		20.78 ± 1.734	19.94 ± 1.259	20.61 ± 1.335	*F* = 1.648	0.203
TMT-A		121 (109.5, 128.75)	123 (108.75, 126.25)	121 (106.5, 136.26)	*Z* = 0.024	0.988
AVLT-immediate recall		18.67 ± 2.169	18.94 ± 2.155	18.67 ± 1.495	*F* = 0.120	0.887
AVLT-delay recall		3.94 ± 1.626	3.44 ± 1.294	3.50 ± 1.383	*F* = 0.651	0.526
AVLT-recognition recall		18.00 ± 1.749	18.06 ± 1.349	18.00 ± 1.029	*F* = 0.009	0.991
BDNF (pg/ml)		134.27 ± 10.79	130.48 ± 12.72	129.48 ± 15.97	*F* = 0.037	0.093
PF4 (ng/ml)		194.17 ± 14.83	203.42 ± 18.52	202.72 ± 15.34	*F* = 0.105	0.096

*N*, sample size; *F*, analysis of variance; χ^2^, chi-square test; *Z*, rank sum test; *p*, probability value for statistical significance. Continuous variables are expressed using mean ± standard deviation, and categorical variables are expressed using frequency (percentage). MoCA, Montreal Cognitive Assessment; AVLT, Auditory Verbal Learning Test, including AVLT-Immediate Recall, AVLT-Delay Recall, and AVLT-Recognition Recall; TMT-A, Trail Making Test-A; BDNF, brain-derived neurotrophic factor; PF4, platelet factor 4.

### 3.2 Comparison of shedding rates in the three groups

During the intervention period, the Tai Chi group had one patient withdraw at the 14th week due to hospital discharge, and another at the 3rd week citing a lack of interest. In the Walking group, one patient withdrew at the 5th week due to hospital discharge, while another withdrew at the 12th week to care for their spouse whose condition had worsened. Within the Control group, two patients voluntarily withdrew, one at the 2nd week and another at the 11th week, both due to hospital discharge. Additionally, two patients in the Control group failed to complete blood sample collection. Fisher’s exact test revealed no statistically significant differences in the number of dropouts among the three groups (*p* > 0.05). Please refer to Table 2 for details.

**TABLE 2:** Comparison of shedding rates in three groups of patients.

Groups	Did not complete motor exercise	Did not complete post-intervention indicator ratings	Total	*P*-value
Tai Chi group	2	0	2	–
Walking group	2	0	2	0.429
Control group	2	2	4	–

### 3.3 Analysis of the results of overall cognitive function in the three groups of patients

#### 3.3.1 Between-group comparisons of overall cognitive functioning outcomes at post-intervention and follow-up in three groups of patients

The overall cognitive functioning scores of the three groups of study participants conformed to a normal distribution with a chi-square variance, and were statistically analyzed using one-way ANOVA. Before the intervention, the MoCA scores of the three groups of patients were consistent at baseline and comparable (*p* > 0.05); after the intervention, the differences in MoCA scores among the three study groups were significant (*p* < 0.05), and after *post hoc* two-by-two comparisons, the differences between the walking group and the control and Tai Chi groups were statistically significant (*p* < 0.05); the differences in MoCA change value scores among the three groups of patients between the intervention and the pre-intervention period were significant (*p* < 0.05), and after *post hoc* two-by-two comparison, the difference between the control group and the Tai Chi group was statistically significant (*p* < 0.05). At the follow-up, the difference in MoCA scores among the three study groups was significant (*p* < 0.05), and after *post hoc* two-by-two comparison, the difference between the control group and the Tai Chi group was statistically significant (*p* < 0.05); the difference in MoCA change value scores between the three groups of patients after intervention and before intervention was significant (*p* < 0.05), and after *post hoc* two-by-two comparison, the difference between the control group and the Tai Chi group was statistically significant (*p* < 0.05). See Table 3.

**TABLE 3:** Between-group comparison of overall cognitive functioning outcomes at post-intervention and follow-up for the three groups of patients (*n* = 54).

	Tai Chi group	Walking group	Control group	*F*	*P*
T1	20.78 ± 1.734	19.94 ± 1.259	20.61 ± 1.335	1.648	0.203
T2	23.06 ± 1.434	21.72 ± 1.274*	20.50 ± 1.383*	15.778	< 0.001
T2–T1	2.279 ± 1.406	1.778 ± 0.832	−0.111 ± 1.656*	16.466	< 0.001
T3	22.17 ± 1.339	21.22 ± 1.353	20.61 ± 1.420*	5.880	0.005
T3–T1	1.389 ± 1.650	1.277 ± 1.127	0.000 ± 0.889*	4.719	0.013

Variables that are normally distributed and have equal variances are described with (X ± S); Differences between groups are analyzed by analysis of variance and multiple comparisons are performed using Dunnett’s test. T1, before intervention; T2, after intervention; T3, Follow-up; *indicates that there is a statistically significant difference with the Tai Chi group according to the *post hoc* pairwise comparison.

#### 3.3.2 Repeated measures ANOVA of overall cognitive function at different time points for all three groups of patients

Taking the MoCA score after intervention and during follow-up as the dependent variable, and taking time, group, and baseline MoCA score as independent variables. At the same time, incorporating the interaction effects of time and group, time and baseline MoCA score, a repeated measures analysis of variance was conducted. The results showed: (1) Grouping factor: The between-group effect of MoCA scores of the three groups of patients was significant (*p* < 0.05), indicating that there were differences in MoCA scores of the three groups of patients at different time points. (2) Time factor: There was no statistically significant difference in the time effect of MoCA scores of the three groups of patients (*p* > 0.05). (3) Baseline MoCA: The MoCA score was affected by the baseline MoCA effect, and the difference was statistically significant (*p* < 0.05). (4) Interaction factor: The interaction effect of time and baseline MoCA score of the three groups of patients was not statistically significant (*p* > 0.05), while the interaction effect of time and group was statistically significant (*p* < 0.05). Therefore, an analysis of the separate effects of time and group is needed. (5) Separate effects: After intervention and during follow-up, there were statistically significant differences in MoCA scores among different groups. After *post hoc* pairwise comparisons, after intervention, there were statistically significant differences between the walking group and the control group compared with the Tai Chi group (*p* < 0.05). During follow-up, there was a statistically significant difference between the control group and the Tai Chi group. However, in different groups, there was no statistically significant difference in MoCA scores after intervention and during follow-up (*p* > 0.05). See Tables 4–6.

**TABLE 4:** Repeated measures ANOVA of overall cognitive function at different time points for the three groups of patients (*n* = 54).

	Time effect	Between-group effect	Baseline MoCA	Interaction effect	
				Time*intergroup	Time*baseline MoCA
*F*-value	0.955	15.839	21.036	3.906	1.393
*P*-value	0.333	< 0.001	< 0.001	0.027	0.244

**TABLE 5:** Separate effects analysis by group (*n* = 54).

Time	Tai Chi group	Walking group	Control group	*F*-value	*P*-value
Post-intervention	23.06 ± 1.434	21.72 ± 1.274*	20.50 ± 1.383*	15.778	< 0.001
Follow-up	22.17 ± 1.339	21.22 ± 1.353	20.61 ± 1.420*	5.880	0.005

*indicates a statistically significant difference between *post hoc* two-by-two comparisons compared to the Tai Chi group.

**TABLE 6:** Analysis of separate effects of time (*n* = 54).

Groups	Post-intervention	Follow-up	*F*-value	*P*-value
Tai Chi group	23.056 ± 1.434	22.167 ± 1.339	3.694	0.063
Walking group	21.722 ± 1.274	21.222 ± 1.353	1.303	0.262
Control group	20.500 ± 1.383	20.611 ± 1.420	0.057	0.813

### 3.4 Specific comparisons of executive function among and within the three groups of patients

#### 3.4.1 Between-group comparisons of executive cognitive functioning outcomes at post-intervention and follow-up in the three patient groups

The executive function scores of the three groups did not conform to a normal distribution and were statistically analyzed using the multiple independent samples non-parametric Kruskal–Wallis H test. Before the intervention, the TMT-A scores of the three groups of patients were consistent at baseline and comparable (*p* > 0.05); after the intervention, the differences in TMT-A scores among the three study groups were significant (*p* < 0.05), and after *post hoc* two-by-two comparisons, the differences between the walking group and the control group and the Tai Chi group were statistically significant (*p* < 0.05); the differences in TMT-A change value scores among the three groups of patients between post-intervention and pre-intervention were significant (*p* < 0.05), and after *post hoc* two-by-two comparison, the difference between the walking group and the control group and the Tai Chi group was statistically significant (*p* < 0.05). At follow-up, the difference in TMT-A scores among the three study groups was significant (*p* < 0.05), and after *post hoc* two-by-two comparisons, the difference between the walking group and the control group and the Tai Chi group was statistically significant (*p* < 0.05); the difference in the TMT-A change value scores between the three groups of patients at follow-up and before intervention was not statistically significant (*p* > 0.05). See Table 7 for specific details.

**TABLE 7:** Between-group comparisons of executive function outcomes at post-intervention and follow-up for the three groups (*n* = 54).

	Tai Chi group	Walking group	Control group	*Z*	*P*
T1	121 (109.5, 128.75)	123 (108.75, 126.25)	121 (106.5, 136.25)	0.024	0.988
T2	102 (98, 107.5)	112.5 (107.75, 116)*	112 (106.75, 117)*	25.13	< 0.001
T2–T1	−20 (−24, −13)	−10 (−13.25, −2.00)*	0.500 (−26, 18.75)*	8.131	0.017
T3	103.5 (100, 105.5)	112 (106.75, 117)*	120 (110, 125.25)*	21.77	< 0.001
T3–T1	−18.5 (−27.5, −3.75)	−6 (−12, 4.25)	−2 (−15.25, 9.5)	5.64	0.060

For variables that do not follow a normal distribution and have equal variances, use M (P25, P75) for description; Differences between groups are analyzed by the non-parametric Kruskal–Wallis H test for independent samples, and multiple comparisons are carried out using the Dunn–Bonferroni method. T1, before intervention; T2, after intervention; T3, at follow-up; *indicates that there is a statistically significant difference with the Tai Chi group according to the *post hoc* pairwise comparison.

#### 3.4.2 Analysis of generalized estimating equations for executive function at different time points for the three groups of patients

Taking the TMT-A score after intervention and during follow-up as the dependent variable, and taking time, group, and baseline TMT-A score as independent variables. At the same time, incorporating the interaction effects of time and group, time and baseline TMT-A score, a generalized estimating equation was constructed. The results showed: (1) Grouping factor: The between-group effect of TMT-A scores of the three groups of patients was significant (*p* < 0.05), indicating that there were differences in TMT-A scores of the three groups of patients at different time points. (2) Time factor: There was no statistically significant difference in the time effect of TMT-A scores of the three groups of patients (*p* > 0.05). (3) Baseline TMT-A: There was no statistically significant difference in the baseline TMT-A effect of TMT-A scores of the three groups of patients (*p* > 0.05). (4) Interaction factor: There was no statistically significant difference in the interaction effects of time and group, time and baseline TMT-A score of the three groups of patients (*p* > 0.05). See Table 8 for details.

**TABLE 8:** Analysis of generalized estimating equations for executive function at different time points for the three groups of patients (*n* = 54).

	Time effect	Between-group effect	Baseline TMT-A	Interaction effect	
				Time*intergroup	Time*baseline TMT-A
Wald*χ^2^* value	0.825	63.333	0.954	1.154	0.921
*P*-value	0.364	< 0.001	0.329	0.562	0.337

### 3.5 Specific comparisons of memory function results between and within the three groups of patients

#### 3.5.1 Between-group comparison of memory function outcomes at post-intervention and follow-up in the three groups

The memory function scores of the three study groups conformed to a normal distribution with a chi-square and were analyzed using one-way ANOVA. Before the intervention, the baseline AVLT immediate memory, AVLT delayed memory and AVLT recollection memory scores of the three groups of patients were consistent and comparable (*p* > 0.05); after the intervention, the differences in AVLT immediate memory, AVLT delayed memory and AVLT recollection memory among the three groups of study subjects were significant (*p* < 0.05), and after *post hoc* two-by-two comparisons, the differences in the walking group and the control group with the Tai Chi group were statistically Significance (*p* < 0.05); the difference in AVLT immediate memory, AVLT delayed memory and AVLT recoginition memory change scores between the three groups of patients after the intervention and before the intervention was significant (*p* < 0.05), and after a *post hoc* two-by-two comparison, the difference between the walking group and the control group and the Tai Chi group was statistically significant (*p* < 0.05). At follow-up, the differences in AVLT immediate memory, AVLT delayed memory, and AVLT recollection memory scores among the three study groups were significant (*p* < 0.05), and after *post hoc* two-by-two comparisons, the differences between the walking group and the control group and the Tai Chi group were statistically significant (*p* < 0.05); and the differences in AVLT delayed memory and AVLT recollection memory change values between the three groups of patients after the intervention and before the intervention were significant (*p* < 0.05), and after *post hoc* two-by-two comparison, the difference between the walking group and the control group and the Tai Chi group was statistically significant (*p* < 0.05); the difference between the AVLT immediate memory change scores of the three groups of patients after intervention and before intervention was significant (*p* < 0.05), and after *post hoc* two-by-two comparison, the difference between the control group and the Tai Chi group was statistically significant (*p* < 0.05). See Table 9 for specific details.

**TABLE 9:** Between-group comparison of memory function outcomes at post-intervention and follow-up in the three groups (*n* = 54).

		Tai Chi group	Walking group	Control group	*F*	*p*
AVLT-immediate	T1	18.67 ± 2.169	18.94 ± 2.155	18.67 ± 1.495	0.120	0.877
	T2	20.72 ± 1.626	18.56 ± 1.977*	18.39 ± 1.420*	10.079	< 0.001
	T2–T1	2.056 ± 2.554	−0.389 ± 3.183*	10.278 ± 1.904*	5.072	0.01
	T3	20.33 ± 1.495	18.94 ± 1.211*	18.50 ± 1.618*	7.814	0.001
	T3–T1	1.667 ± 2.029	0.000 ± 2.425	−0.167 ± 2.479*	3.437	0.040
AVLT-delay	T1	3.94 ± 1.626	3.44 ± 1.294	3.50 ± 1.383	0.651	0.526
	T2	6.39 ± 0.979	3.83 ± 0.985*	3.17 ± 0.985*	53.894	< 0.001
	T2–T1	2.444 ± 1.617	0.389 ± 1.501*	−0.333 ± 1.495*	15.795	< 0.001
	T3	5.78 ± 0.808	3.33 ± 0.970*	3.00 ± 0.840*	53.994	< 0.001
	T3–T1	1.833 ± 1.724	−0.111 ± 1.367*	−0.500 ± 1.339*	12.721	< 0.001
AVLT -recognition	T1	18.00 ± 1.749	18.06 ± 1.349	18.00 ± 1.029	0.009	0.991
	T2	21.22 ± 1.263	18.83 ± 1.339*	18.5 ± 2.121*	15.092	< 0.001
	T2–T1	3.222 ± 1.865	0.778 ± 1.927*	0.500 ± 2.332*	9.493	< 0.001
	T3	20.83 ± 1.618	18.50 ± 1.249*	19.41 ± 1.848*	11.497	< 0.001
	T2–T1	2.833 ± 1.757	0.444 ± 1.947*	0.889 ± 1.967*	8.110	0.001

Variables that are normally distributed and have equal variances are described with (X ± S); Differences between groups are analyzed by analysis of variance and multiple comparisons are performed using Dunnett’s test. T1, before intervention; T2, after intervention; T3, follow-up; *indicates that there is a statistically significant difference with the Tai Chi group according to the *post hoc* pairwise comparison.

#### 3.5.2 ANOVA of repeated measures of memory function at different time points in the three groups

Taking the AVLT scores after intervention and during follow-up as the dependent variable, and taking time, group, and baseline AVLT score as independent variables. At the same time, incorporating the interaction effects of time and group, time and baseline AVLT score. According to different measurement times of memory cognitive function, repeated measures analysis of variance was conducted on AVLT immediate, AVLT delayed, and AVLT recognition, respectively. The results showed: (1) Grouping factor: The between-group effect of AVLT scores of the three groups of patients was significant (*p* < 0.05), indicating that there were differences in AVLT immediate, AVLT delayed, and AVLT recognition scores of the three groups of patients at different time points. (2) Time factor: There was no statistically significant difference in the time effect of AVLT scores of the three groups of patients (*p* > 0.05). (3) Baseline AVLT: The AVLT delayed score was affected by the baseline AVLT effect, and the difference was statistically significant (*p* < 0.05). There was no statistically significant difference in the influence of baseline AVLT effect on AVLT immediate and AVLT recognition scores (*p* > 0.05). (4) Interaction factor: The interaction effects of time and group, time and baseline AVLT score of the three groups of patients were not statistically significant (*p* > 0.05). See Table 10.

**TABLE 10:** Analysis of generalized estimating equations for memory-knowledge function at different time points for the three groups of patients (*n* = 54).

Item		Time effect	Intergroup effect	Baseline AVLT	Interaction effect	
					Time*intergroup	Time*baseline AVLT
AVLT immediate	*F*-value	0.205	12.588	0.230	0.852	0.221
	*P*-value	0.653	< 0.001	0.633	0.433	0.640
AVLT delay	*F*-value	0.664	99.065	6.030	0.593	0.007
	*P*-value	0.419	< 0.001	0.018	0.557	0.932
AVLT -recognition	*F*-value	0.553	17.497	1.310	1.341	0.500
	*P*-value	0.461	< 0.001	0.258	0.271	0.483

### 3.6 Analysis of serum BDNF results in three groups of patients

**FIGURE 2:**
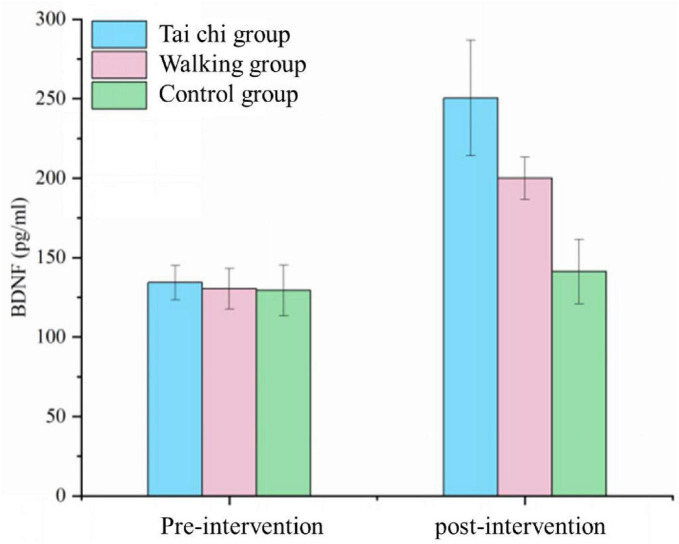
Results of serum BDNF in three groups of patients (*n* = 52).

The serum BDNF scores of the three study groups followed a normal distribution and exhibited homogeneity of variance, and were therefore compared using analysis of variance (ANOVA). At baseline, the serum BDNF levels among patients in the Tai Chi group, walking group, and control group were 134.27 ± 10.79 pg/ml, 130.48 ± 12.72 pg/ml, and 129.48 ± 15.97 pg/ml, respectively. These baseline differences in serum BDNF levels between the three groups were comparable (*p* > 0.05). Following the intervention, the serum BDNF levels increased to 250.44 ± 36.37 pg/ml in the Tai Chi group, 199.96 ± 13.35 pg/ml in the walking group, and remained at 141.25 ± 20.26 pg/ml in the control group. A statistically significant difference in serum BDNF levels was observed among the three groups post-intervention (*p* < 0.05). *Post hoc* pairwise comparisons revealed a significant difference between the control group and the Tai Chi group (*p* < 0.05). Additionally, the changes in serum BDNF levels from pre- to post-intervention were statistically significant across all three groups (*p* < 0.05), with a significant difference noted specifically between the control group and the Tai Chi group upon *post hoc* analysis (*p* < 0.05). See Table 11 and Figure 2 for details.

**TABLE 11:** Analysis of serum BDNF results in three groups of patients (*n* = 52).

	Tai Chi group	Walking group	Control group	*F*	*P*
T1	134.27 ± 10.79	130.48 ± 12.72	129.48 ± 15.97	0.037	0.093
T2	250.44 ± 36.37	199.96 ± 13.35	141.25 ± 20.26*	4.436	0.017
T2–T1	116.17 ± 30.46	69.48 ± 12.38	11.77 ± 12.34*	6.161	0.004

Variables that are normally distributed and have equal variances are described with (X ± S); Differences between groups are analyzed by analysis of variance and multiple comparisons are performed using Dunnett’s test. T1, before intervention; T2, after intervention. CV = 9.56. *indicates a statistically significant difference compared with the Tai Chi group (*post hoc* pairwise comparisons).

### 3.7 Analysis of serum PF4 results in three groups of patients

**FIGURE 3:**
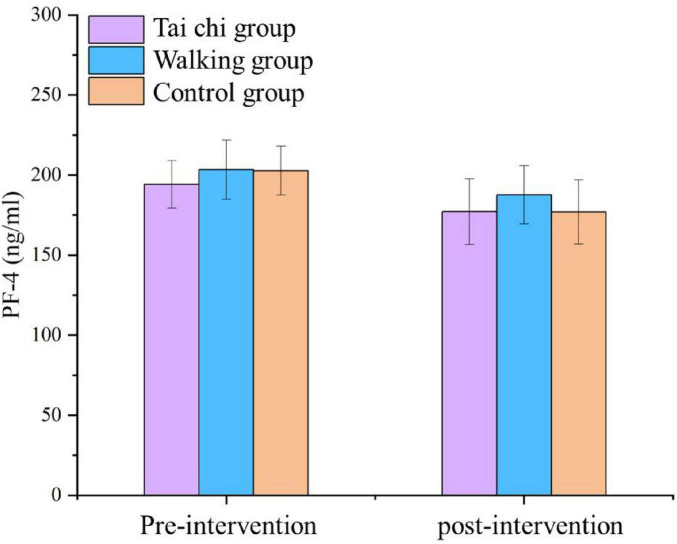
Results of serum PF4 in three groups of patients (*n* = 52).

The serum PF4 scores of the three study groups were normally distributed and homogeneous, and were analyzed by ANOVA for comparison. The difference in serum PF4 levels at baseline among the three groups was comparable (*p* > 0.05). The serum PF4 level was 177.14 ± 20.50 ng/ml in the Tai Chi group, 187.67 ± 18.17 ng/ml in the walking group, and 176.94 ± 20.08 ng/ml in the control group of patients after the intervention. there was no statistically significant difference between the three groups of patients in terms of their serum PF4 levels after the intervention (*p* > 0.05). The difference between the serum PF4 levels of the three groups of patients after intervention and before intervention was not statistically significant (*p* > 0.05). See Table 12 and Figure 3.

**TABLE 12:** Analysis of serum PF4 results in three groups of patients (*n* = 52).

	Tai Chi group	Walking group	Control group	*F*	*P*
T1	194.17 ± 14.83	203.42 ± 18.52	202.72 ± 15.34	0.105	0.096
T2	177.14 ± 20.50	187.67 ± 18.17	176.94 ± 20.08	0.901	0.909
T2–T1	−42.09 ± 21.95	−19.03 ± 20.19	6.03 ± 19.23	1.332	0.273

Variables that are normally distributed and have equal variances are described with (X ± S); Differences between groups are analyzed by analysis of variance and multiple comparisons are performed using Dunnett’s test. T1, before intervention; T2, after intervention. CV = 10.28.

## 4 Discussion

### 4.1 The effect of Tai Chi on the overall cognitive function of MCI patients

The results presented in Tables 3–6 reveal that, following the intervention, Tai Chi can enhance the overall cognitive function of MCI patients in comparison with walking and routine activities. During the follow-up, Tai Chi is also capable of improving the overall cognitive function of MCI patients when compared with routine activities. When compared with walking and routine activities, the difference in the overall cognitive function score of Tai Chi from before to after the intervention is significant (*p* < 0.05), and in comparison with routine activities, the difference in the overall cognitive function score of Tai Chi from before the intervention to during the follow-up is also significant (*p* < 0.05). This indicates that the MoCA score of the subjects in the Tai Chi group is the highest upon the completion of the intervention, followed by the walking group, and subsequently, it gradually declines. Meanwhile, the range of change in the MoCA score of the subjects in the control group is relatively small.

However, the improvement in the overall cognitive function during the follow-up does not achieve statistical significance. Some studies have confirmed (Cui et al., 2021) that long-term Tai Chi may protect cognitive function and promote cognitive flexibility by improving the plasticity of functional brain networks. The human brain is a vast network consisting of various regions, each responsible for distinct functions (Fam et al., 2020). As individuals age, there is often a disruption in connectivity between these brain regions (Yan and Rein, 2022). Studies have shown that multimodal interventions, such as cognitive training, Tai Chi, and group counseling, can effectively enhance functional connectivity between the medial prefrontal cortex and the medial temporal lobe. Furthermore, the strength of this connectivity has been positively correlated with improvements in cognitive function (Tao et al., 2017). Hui et al. (2016) monitored the functional connectivity of different brain regions in Tai Chi practitioners and routinely active controls during rest and exercise states, and they found that Tai Chi activated the prefrontal cortex, motor cortex, and occipital cortex, and improved their connectivity in terms of musculogenic activity, the sympathetic nervous system, and the metabolic activity of endothelial cells. In addition, Tai Chi increases connectivity between the left middle frontal gyrus and the left superior parietal lobule, the posterior cingulate gyrus cortex, and the left prefrontal cortex and the right sensorimotor areas (Wang and Lu, 2022). Other studies have shown that Tai Chi also facilitates the establishment of cognitive reserve, increases synaptic nerves, prompts the brain to continuously produce new and functional neurons, increases the capacity of cognitive reserve (Gould et al., 1999), prevents age-related neurocognitive decline, reduces neurological dysfunction, and delays the onset and progression of MCI to AD (Stern, 2012). Therefore, the beneficial effects of Tai Chi on cognitive function in patients with MCI may be realized by altering the connectivity of brain networks and increasing cognitive reserve.

Our study also found that the improvement effect on cognitive function in MCI patients tended to decrease with the cessation of Tai Chi intervention. Related studies have also found that after the intervention, even though Tai Chi had a positive effect on overall cognitive function, its beneficial effects gradually disappeared after cessation of training, and only those who continued to receive Tai Chi maintained their cognitive function at the previous level (Li et al., 2023). Therefore, for MCI patients with conditional physical conditions, prolonged Tai Chi can be performed to delay cognitive decline.

### 4.2 Effects of Tai Chi on executive function in MCI patients

The results presented in Tables 7, 8 demonstrate that, in comparison with walking exercise and daily activities, Tai Chi is capable of enhancing the executive function of MCI patients after the intervention and throughout the follow-up period. Specifically, when compared to the status before the intervention, the executive function of MCI patients in the Tai Chi group has exhibited significant improvements both after the intervention and during the follow-up. Meanwhile, compared to the pre-intervention status, the executive function of patients in the walking group tends to improve after the intervention. This suggests that both Tai Chi and walking exercise can improve the executive function of MCI patients, yet the influence of Tai Chi on the executive function of MCI patients is more enduring.

A systematic review and meta-analysis found that the advantages of Tai Chi were more significant in improving executive functions such as processing speed, attention, and working memory, compared with inactivity or other exercises (walking, resistance, and flexibility exercises) (Wayne et al., 2014). A pilot study also showed that 24 weeks of 10-pattern Tai Chi had a positive effect on executive function in older adults with MCI (Sungkarat et al., 2018). An RCT by Lam (Lam et al., 2011) assessing the effects of 24-pattern and 10-pattern Tai Chi on the executive function components showed that Tai Chi had a significant improvement on the task-switching component of executive function. The present study is consistent with the findings of the above scholars.

Tai Chi requires visuospatial orientation through learning a series of movements, including motor recall, switching, and multi-segmental movements, and focusing on inhibiting disturbances in the surrounding environment, which is beneficial to increasing brain volume and cortical thickness (Wei et al., 2013). Mortimer et al.’s (2012) study showed that 40 weeks of Tai Chi increased whole-brain volume by 0.47%, which was significantly higher than that of either the walking group or the group doing activities of daily living group. Primarily, Tai Chi increased brain volume by promoting an increase in gray matter volume in the left middle occipital gyrus, left superior temporal gyrus, and right middle temporal gyrus (Cui et al., 2021) and increased cortical thickness in brain regions closely related to executive functions (precentral gyrus, middle frontal sulcus, superior temporal gyrus, and medial occipito-temporal sulcus) (Wei et al., 2013).

Thus, Tai Chi improves executive function in MCI patients more than walking exercise or regular activities. It may be due to the fact that Tai Chi increases brain volume and cortical thickness more.

### 4.3 The effect of Tai Chi on memory function in MCI patients

From Tables 9, 10, it can be found that Tai Chi improves the memory function of MCI patients after the intervention and during the follow-up compared to walking exercise and regular activities. Compared with the pre-intervention period, the memory function of MCI patients in the Tai Chi group improved significantly at post-intervention and follow-up. This suggests that Tai Chi can significantly improve the memory function of MCI patients, but there is a tendency for the memory function to slowly decline as the exercise ends.

A randomized controlled trial from Thailand reported that the increase in delayed recall scores was more pronounced in older adults with MCI who practiced the 10-posture Tai Chi than in the control group (Sungkarat et al., 2018). an RCT in China found a significant increase in memory scores in the Tai Chi group compared to the other three groups (walking, socializing, or no intervention) (Mortimer et al., 2012). another RCT also showed that MCI participants who performed 24-posture simplified Tai Chi compared to performing stretching activities showed significant improvement in delayed recall (Lam et al., 2012). the results of the present study were similar to the above studies, further confirming the improvement of memory function by Tai Chi.

Oxyhemoglobin is a reliable indicator of changes in regional cerebral blood flow and can be used to reflect the degree of prefrontal cortex activation during exercise (Perrey, 2008), and decreased prefrontal cortex activation may lead to memory loss (Uemura et al., 2016). Lu et al. (2016) monitored oxygenated hemoglobin in Tai Chi exercisers and cyclists, and found that Tai Chi exercisers showed a greater increase in oxygenated hemoglobin and total hemoglobin levels indicating that prefrontal neuron activation is higher during Tai Chi than cycling. Therefore, Tai Chi can enhance memory function by activating the prefrontal cortex.

### 4.4 Effect of Tai Chi on serum BDNF in MCI patients

Based on the combination of Table 11 and Figure 1, it was found that serum BDNF levels were significantly higher in MCI patients in the Tai Chi group compared with the walking group and the control group (*P* < 0.05). Serum BDNF levels in MCI patients in both the Tai Chi and walking groups were higher after the intervention than before (*P* < 0.05). This suggests that both Tai Chi and walking exercise can increase serum BDNF levels in patients with MCI, but Tai Chi can increase serum BDNF concentration in patients with MCI, whereas walking exercise cannot.

Many studies on exercise intervention for cognitive function, the ending indexes are mostly based on scale scores and lack blood-based indexes that respond to changes in cognitive function. Brain-derived neurotrophic factor (BDNF), a member of the neurotrophic factor family, was initially found in the brain and is widely expressed in the hippocampus and cortex, where it promotes cell survival and neurite growth through a cascade of tyrosine kinase receptor-induced molecular signaling (Mattson et al., 2004), as well as synaptic plasticity and neuronal development (Barde et al., 1982). There is evidence in aged rodents and primates that BDNF reverses neuronal atrophy (Nagahara et al., 2009), increases the number of synapses (Tyler and Pozzo-Miller, 2001), and promotes neurodevelopment (Bergami et al., 2008).

Animal studies have shown that physical exercise increases BDNF expression in hippocampal and cortical regions (Uysal et al., 2015), and an experimental study found that 6 months of aerobic exercise increased serum BDNF levels in patients with MCI (Baker et al., 2010). Whereas the increase in serum BDNF has been associated with the effect of exercise in improving cognitive function (Griffin et al., 2011), the results of another experimental study also showed that aerobic exercise-induced up-regulation of serum BDNF was associated with an increase in hippocampal volume and an improvement in memory function (Erickson et al., 2011), and Sungkarat et al. (2018). demonstrated that 6 months of Tai Chi practice was effective in up-regulating plasma BDNF while improving the memory and thinking function in elderly MCI patients’ memory and thought transitions, and similar phenomena were observed in another 10-week Tai Chi intervention study (Solianik et al., 2021). Consistent with these findings, the present study also found that Tai Chi increased serum BDNF concentrations while improving overall cognitive, executive, and memory functions in MCI patients.

Tai Chi is a mind-body exercise with integrated cognitive and motor coordination, while mentally focused psychotherapy activates neurons and prompts the release of glutamate from presynaptic endings, increasing intracellular Na^+^ and Ca^2+^, and Ca^2+^ activates the production of BDNF via N-methyl-d-aspartate receptor endocytosis in dendrites (Damirchi et al., 2018). Consequently, the improvement in cognitive function observed in MCI patients who practice Tai Chi may be attributed to the increase in serum BDNF levels, which is likely due to the stimulation of cellular mechanisms associated with neurotrophic factors.

### 4.5 Effect of Tai Chi on serum PF4 in MCI patients

Platelets are small anucleated blood cells that store bioactive factors in specialized cytoplasmic compartments (van der Meijden and Heemskerk, 2019), and are of interest for their powerful and surprising reservoirs of anti-inflammatory, neurotrophic, and antioxidant molecules, and different forms of platelet activation release their inclusions selectively and releasably depending on the specific situation in response to environmental stimuli such as exercise, tissue injury, or stress; thus, different forms of platelet activation can produce fundamental biological effects ranging from hemostasis to neurogenesis (Leiter et al., 2019), including postoperative healing (Marx et al., 1998), treatment of musculoskeletal injuries and osteoarthritis (Dai et al., 2017; Kavadar et al., 2015), and rejuvenation of the skin (Ozcelik et al., 2016). PF4 is a specific protein synthesized by platelets, and the neuroprotective effects of PF4 have been demonstrated in mouse models of traumatic brain injury (Nebie et al., 2021), amyotrophic lateral sclerosis (Gouel et al., 2022), and Parkinson’s disease (Chou et al., 2017). The idea that PF4 may be a messenger of brain health was supported by a study by Leiter et al. (2019) who found that platelets in young mice were activated after a short period of acute exercise (4 days) and that the subsequent release of PF4 from platelets increased proliferation of hippocampal precursors and facilitated neuronal differentiation. two more recent studies have found that PF4 attenuates age-related hippocampal neuroinflammation that triggered molecular changes related to synaptic plasticity and improved cognitive performance in aged mice (Schroer et al., 2023) and increased cognitive performance in young and aging mice (Park et al., 2023). Immediately following this, a study by Leiter et al. (2023) clarified the activation response of exercise on platelets in young and aged mice and determined that PF4 released from exercise-induced activated platelets rejuvenated hippocampal neurogenesis and cognitive function in aged mice.

From Table 12 and Figure 2, we can observe that there is no significant alteration in the PF4 levels of the three groups of MCI patients after the interventions of Tai Chi and walking exercise when pairwise comparisons are performed, and the difference is not statistically significant (*P* > 0.05). Similarly, when comparing the serum PF4 levels of the Tai Chi group and the walking group after intervention with those before intervention, there is also no significant change, and the difference is not statistically significant (*P* > 0.05). Our study is the first exploratory trial that utilizes PF4 as a serum concentration indicator for detecting MCI patients after exercise (Tai Chi or walking exercise). however, unfortunately, the concentration of serum PF4 levels in this study did not show an increase in the concentration of serum PF4 levels due to the intervention of the exercise, but instead, there was a trend of a slight decrease in the levels of PF4 with the completion of the intervention; suggesting that the effect of Tai Chi or walking exercises on the serum PF4 concentrations in MCI patients had no effect because, with age, PF4 in human and non-human primate plasma gradually decreases (Wang H. et al., 2019). In our analysis, both Tai Chi and walking exercise are chronic exercises of low to moderate intensity, which may not be sufficient to induce platelet activation to release more PF4 factor. Therefore, our study did not yield evidence that Tai Chi increased serum PF4 levels in patients with MCI.

### 4.6 Limitations of the study

(1) Single-center experimentation: This study was conducted exclusively within a single healthcare integration facility in Chengdu, potentially encountering obstacles when generalizing the findings to the broader elderly population afflicted with MCI, thereby limiting its universal applicability.
(2) Insufficient sample size: Constrained by both human and material resources, the study enrolled a relatively small cohort of 54 patients. This small sample size may undermine statistical power and elevate the uncertainty surrounding the interpretation of results.
(3) Limited biomarker selection: The study focused solely on exercise-related biomarkers, namely serum brain-derived neurotrophic factor (BDNF) and platelet factor 4 (PF4), excluding the inclusion of characteristic biomarkers of Alzheimer’s disease (AD) such as β-amyloid (Aβ) and phosphorylated tau (*p*-tau). This limitation may restrict our in-depth understanding of the underlying disease mechanisms.
(4) Potential bias risk: The lack of an active control group in the study design could compromise the accuracy of experimental outcomes, as the absence of a proper comparative standard introduces the risk of bias into the results.

## 5 Conclusion

In this work, a 12-week trial of Tai Chi intervention was conducted in MCI patients to investigate the clinical efficacy of exercise intervention in MCI patients by collecting patients’ scale scores of overall cognitive function, executive function, and memory function, and the changes of serum BDNF and serum PF4 levels before and after the trial at pre-test, post-test, and follow-up. The results of the study showed that Tai Chi improved the overall cognitive function, executive function, and memory function of MCI patients, and the mechanism may be related to increasing the expression of serum BDNF levels. The positive effects of Tai Chi on serum PF4 levels in MCI patients have yet to be verified. This study provides research evidence for the improvement of cognitive function in MCI by Tai Chi, and provides a theoretical basis for its clinical application and promotion.

## Data Availability

The raw data supporting the conclusions of this article will be made available by the authors, without undue reservation. Requests to access the datasets should be directed to Y-XH, heyaoxi202110@126.com.
